# Cases in Midwifery

**Published:** 1828-06

**Authors:** Richard Long

**Affiliations:** Surgeon to the Arthurstown Dispensary.


### MIDWIFERY.

*Cases in Midwifery.* By RICHARD LONG, M.D. Surgeon to the Arthurstown Dispensary.

THERE is, perhaps, no branch of surgical science in which promptness of decision and firmness of purpose are more required than midwifery. In submitting the following cases to the public, I have no new-fangled theory to advance or bolster up. If, in the moment of danger, a recollection of them may inspire hope where all appears hopeless, and give nerve to the operator where every thing would point out despair, my wishes will be fully accomplished.

In April, 1826, I was called to Mrs. F., aged thirty-four years, who was in her fourth accouchement, her former labours having been uncommonly favorable and quick. In the present instance, she had been in labour four days ; there had been an arm presentation ; the arm was removed, the thorax perforated, its contents evacuated, and, as it afterwards appeared, the crotchet had been passed through the thorax, and fixed between the first and second cervical vertebræ. In this position the unremitting exertions of the gentleman in attendance, a powerfully strong man, had been used for hours to accomplish delivery without effect. On my visit, the uterine action continued powerfully strong, so as to preclude any attempt at turning with safety; it was also clear that delivery could not be accomplished while the body and head of the child remained in the present posture ; I therefore sought for the other arm, which was removed, and immediately the remaining portion of the body was expelled with violence; but, to my great dismay, the head was separated, and remained within the uterus. A moment's hesitation, and all was lost. Before the uterus had time to contract, I introduced my hand, and firmly fixed the head against the side of the uterus, my colleague at the same time steadying it by external pressure: the head was then perforated, and extraction accomplished by the crotchet more easily than I could have imagined.

In June, 1827, I was again called to the same patient, who had been some hours before delivered, naturally, of a still-born child; the placenta was retained without hemorrhage. On examination, a firm hour-glass contraction was found in the centre of the uterus : this, however, was not the cause of the placenta being retained, as it was found firmly adherent near the fundus uteri, and required a constant and tedious exertion with the fingers to effect its separation piece by piece. The part of the uterus to which the placenta adhered had a grisly semi-cartilaginous feel, such as I have before found the uterus to acquire when injury had been sustained in former labours.

I was informed that this woman appeared to go on well for several days after her confinement, but, from some mismanagement, peritoneal inflammation was produced, by which she was carried off.

Practically speaking, the notions entertained of the possibility of unassisted delivery being accomplished in arm presentations, either by spontaneous evolution of the fœtus, or by nates and shoulder being protruded together, have been productive of much injury.

In arm presentations, decision is every thing: if assistance is sought where the pelvis is at all tolerably formed, the child can be always safely turned, no matter how violently the uterus acts, provided the shoulder and chest are not wedged in the strait of the pelvis. On the other hand, if the shoulders and breast of the child are forced down, and the capacity of the pelvis is narrow, or only of ordinary dimensions, I conceive it is a useless sacrifice of life to allow an unfortunate woman to exhaust herself by protracted pains, under the expectation of evolution of the fœtus, or birth by doubling of the body.

*Uterine Hemorrhage.*—In November, 1827, I was requested to visit Mrs. W., aged thirty-nine years, who had been delivered two hours before of her eighth child. I found her to all appearance dead: there was no perceptible pulsation or breathing, except a slight convulsive heaving of the chest; the eyelids were half opened, and the eyes turned up, as in instances of recent death; the lips were colourless, the face cold and clammy, and the extremities quite cold. On examination, the uterus was found flaccid and uncontracted, and the woman drenched in blood. The placenta had been removed.

The hand was instantly passed into the body of the uterus, and, by frequent friction of its inner surface, muscular contraction was excited. The nostrils were stimulated by hartshorn, and warm wine and hartshorn were poured down the throat, apparently without any effort of deglutition by the patient. Warmth was applied to the extremities, and a large sheet, quadrupled, and wetted in the coldest spring water, was placed over the abdomen. For some hours, the success of those means, although steadily persevered in, appeared extremely doubtful. As the uterine action, however, was restored, the vital spark appeared to rekindle, and animation was slowly but perfectly established. After several weeks of suffering from extreme debility, the patient has been able to resume the care of her domestic affairs.

In the practical application of the history of the foregoing case, it may be asked what would have been the result had the attention been directed to the administration of stimuli and the transfusion of blood, instead of endeavouring to remove the debilitating cause by mechanically exciting uterine contraction?

*Arthurstown, co. Wexford; March 7,* 1828.

